# An assessment for diagnostic and therapeutic modalities for management of pediatric Iron defficiency Anemia in Saudi Arabia: a crossectional study

**DOI:** 10.1186/s12887-019-1704-3

**Published:** 2019-09-05

**Authors:** Hadi J. Al Sulayyim, Ali Al Omari, Motasim Badri

**Affiliations:** 1 Department of Epidemiology and Biostatistics, College of Public Health and Health Informatics, King Abdulaziz Medical City, Ministry of National Guard Health Affairs, King Saud bin Abdulaziz University for Health Sciences, Riyadh, Kingdom of Saudi Arabia; 2 Division of Pediatric, Department of Oncology, King Abdulla Specialized Children Hospital, King Abdulla International Medical Research Centre, Riyadh, Saudi Arabia

**Keywords:** Iron deficiency anemia, Treatment, Diagnosis, Assessment, Pediatric

## Abstract

**Introduction:**

Iron deficiency anemia (IDA) is a global public health issue that affect more than 2 billion individuals worldwide. However evidence for optimal management of IDA is lacking.

**Methods:**

To assess the diagnostic criteria and therapeutic modalities for pediatric IDA employed by physicians in a major public healthcare facility in Riyadh, a validated questionnaire including demographic data and patient case-scenarios related to diagnosis and treatment of IDA was employed. Robust regression analysis was used to identify factors associated with overall score of participants.

**Results:**

Of the 166 physicians surveyed 147(88.6%) were included in the study. Wide variability was observed in IDA diagnosis and therapy practises. For nutritional IDA, only 15.6% recommended no other laboratory tests in addition to CBC. The majority preferred treatment with ferrous sulfate (77.6%) divided into two doses (57.1%), but the total daily elemental iron doses varied widely from 2 to 6 mg/kg. For intravenous iron, 42.9% recommended iron dextran, 32.7% iron sucrose, and 13.4% would continue oral iron. Of all assessed factors, median score was significantly highest in pediatric hematologists compared with pediatricians, family medicine specialists and GPs; *p* = 0.007, and those work in tertiary care compared with those in primary care; *p* = 0.043. However, in multivariate robust regression analysis, overall score was only significantly associated with professional qualification [pediatric hematologist **β =** 13.71,95%CI 2.48–24.95, *p* = 0.017; pediatrician **β =** 1.77,95%C (− 6.05–9.59, *p* = 0.66; family medicine **β =** 2.66,95%CI-4.30-9.58, *p* = 0.45 compared with general practitioner].

**Conclusion:**

Wide variations exist among physicians in diagnosis and treatment of pediatric IDA. Intervention programs and national guidelines are urgently needed.

## Introduction

Globally, Iron Deficiency (ID) is the most common nutritional deficiency. It affects more than 2 billion individuals in the world. Iron deficiency anemia (IDA) accounts approximately for 50% of all anemia cases in the world [1]. In developed countries the prevalence of IDA amount to 1–18% [2], compared with 30–51% in developing countries [3]. Pregnant women, infants aged 4 months to 2 years, young female, vegetarian people, and school-aged children particularly are at increased risk of IDA [4]. Poor dietary and inadequate knowledge among young people are also implicated.

IDA is a consequence of loss of iron, which plays a significant role in normal RBCs synthesis [5]. The process of RBCs production, haematopoiesis, requires iron. About two-thirds of the human body’s iron is contained in Hb [6]. Iron is specifically important for the central nervous system development, particularly at the first 2 years of life. Approximately 80% of the amount of iron in newborns, up to 1 year of age, is accreted during the last 3 months of pregnancy.

A study conducted in the United States found that 9% of infants aged 1–2 years, and 9–10% of teenagers had IDA [7]. In some Arabic countries, the prevalence of IDA among infants was above 70% [8]. However, in the Kingdom of Saudi Arabia (KSA) IDA prevalence among infants who attended well-baby clinics amounted to 52% [9]. A cross-sectional study conducted in Jeddah estimated an IDA prevalence of 20.5% among school children [10]. Another study conducted in a northwestern region of KSA found that 49% of infants aged 6 months to 2 years suffered from IDA [11]. A study conducted among school girls aged between 7 and 14 years in Riyadh reported an IDA prevalence of 26% [12]. Many studies examining the relationship between IDA and neurological/behavioral developments in children found significant associations between IDA and neurological/behavioral consequences [12–14].

The American Academy of Pediatrics (APP) recommends that children at 1 year of age should undergo comprehensive screening for anemia. This includes determining Hb concentration and assessing IDA risk factors. These risk factors include history of low birth weight, continued breastfeeding after 4 months of birth without iron supplementation, decreased socioeconomic status, lead exposures, foods not rich with iron. If the IDA risk factors are noticed in children between one and 3 years, prompt screening should be conducted [15, 16].

However, variability still exists in diagnosis of IDA. Many patients undergo unnecessary tests in addition to the complete blood count (CBC) [17]. Some reports emphasized use of hematological parameters such as serum transferrin, serum ferritin (SF), transferrin receptor 1 (TfR1), morphology and number of RBCs and Hb concentration. Iron status is commonly assessed by SF, while anemia is also commonly assessed by Hb [18]. SF is considered a sensitive test for Iron stores. The concentration of SF less than 12 g/l is considered indicative of ID [19]. However, SF concentration may increase in the presence of some health conditions, such as malignancy, infection, hepatic disease or chronic inflammation. Therefore, it has been recommended that tests not affected by these health conditions, such as C-reactive protein (CRP), Reticulocyte Hemoglobin Content (CHr) or TfR1 must be added as a diagnostic test when the patient is diagnosed with any of these conditions [20]. Although CHr has been shown to represents a strong biomarker for pediatric ID [21], however, it has been argued that the CHr test cannot differentiate between IDA and thalassemia, and TfR1 cannot differentiate between IDA and hemolytic anemia. In addition, the TfR1 test is not yet widely available in many countries [21].

Of concern, current therapeutic intervention practice is largely based more on experience than evidence [22]. Appropriate iron therapy requires an appropriate dose for enough time duration [23]. An increased Hb of 1 gram per dl after 30 days of medication is considered a proper response to therapy and confirms IDA diagnosis. Current available medical literature suggests that 3–6 mg/kg/day of iron is an efficient IDA treatment [24, 25]. However, despite being the overwhelming preference for pediatric hematology/oncology specialists, no evidence is available yet to support the choice of iron dosage of 6 mg/kg/day in children. Many studies have shown success with low dosages and that higher dose might result in increased adverse events (AEs) and poor adherence [26].Typically, treatment is taken orally, but treatment through parenteral route might be used in IDA patients who cannot tolerate oral route; for instance, those diagnosed with gastrectomy, bariatric surgery, and small intestine surgery [27].

In summary, evidence-based guidelines for optimal management of IDA are lacking. This is because diagnosis of IDA often is challenging, and a highly sensitive and specific diagnostic test currently is not available. In addition, despite the fact that IDA treatment consists initially of oral iron supplement, there is no consensus on the schedule of dosage and the total period of therapy [22].

This study aims to document and analyze the diagnostic criteria and therapeutic modalities employed by pediatricians in healthcare facilities affiliated with the Ministry of National Guard, Riyadh, KSA.

## Patients and methods

An anonymous cross-sectional survey was conducted at healthcare facilities affiliated with the Ministry of National Guard Health Affairs in the central region of KSA between 16 April and 19 October 2017. These healthcare facilities included primary care [Iskan Clinic, Health Care Specialty Center (HCSC), National Guard Comprehensive Specialized Clinic (NGCSC), Dirab Clinic, and Price Bader primary care clinic (PBRCC)] and tertiary (KASCH). All general practitioners (GP), family medicine physicians, pediatricians, and pediatric hematologists working at these facilities were invited to participate in the study. The purpose of the study was explained in details and those willing to participate signed a formal consent form.

A validated English version questionnaire was used to assess the diagnostic criteria and therapeutic modalities for pediatric IDA employed by each respondent [22]. The questionnaire included two parts. The first part included demographic data and the second part of questionnaire included two case scenarios. The case scenario was presented and the respondent was asked to identify the appropriate diagnostic tools and the optimal therapeutic interventions of his/her preference.

The responses were evaluated based on published evidence for diagnosis and treatment of IDA.^22, 24, 28–30,^ An overall score (for diagnosis and treatment, combindly) was calculated for each participant based on his/her response to all items in the questionnaire. The overall score was 19.

### Statistical analysis

Data were presented as proportions (%) or median (inter-quartile range) and compared using the χ^2^ - test, Mann-Whitney test or Kruskal-Wallis Test, as appropriate. The Robust regression analysis method was used to identify variables associated with score. These variables include socio-demographic, professional qualification, professional rank, type of current work setting, years in practice since fellowship, number of co-worker pediatricians and availability of fellowship program at work. Variables found significant in Robust univariate analyses were included in the final multivariate model. All tests were two-sided and a *p*-value < 0.05 was considered significant. The statistical packages IBMSPSS (release 20) and Stata (StataCorp, Texas, USA, version 15) were used for data management and analysis.

## Results

Of the 166 surveyed, 19 participants were excluded because they did not complete the full questionnaire. Therefore, the final number of participants included in the study was 147(88.6%).

Socio-demographic and professional characteristics of the participants are shown in Table 1. Median age was 39 [28–43] years and 50.3% were Males. Most of the respondents were pediatricians 77(52.4%) and only 6 (4.1%) were associate consultants. 48.3% of participants reported their current work setting was a primary care center and 51.7% a tertiary care center. Approximately 50% of the participants reported that more than 10 pediatricians’ practice at their centers and 60.5% reported that there was a pediatric fellowship associated with their centers; Table 1.

**Table 1:** Socio-demographic and professional characteristics of the participants

Characteristic		*N*	%
Age, Median (Q1-Q3)		39.0	[28–46]
Gender	F	73.0	49.7
	M	74.0	50.3
Professional Qualification	GP	50.0	34.0
	Family medicine	12.0	8.2
	Pediatrician	77.0	52.4
	Pediatric Hematologist	8.0	5.4
Professional Rank	Assistant Consultant	22.0	15.0
	Associate Consultant	6.0	4.1
	Consultant	36.0	24.5
	staff physician	48.0	32.7
	Resident	33.0	22.4
Type of current work setting	Primary Care	71.0	48.3
	Tertiary Care	76.0	51.7
Years in practice since fellowship	0–5 years	58.0	39.5
	6–10 years	29.0	19.7
	11–15 years	16.0	10.9
	> 15 years	27.0	18.4
No. of co-worker pediatricians	1–2 physicians	47.0	32.0
	3–5 physicians	19.0	12.9
	6–10 physicians	7.0	4.8
	> 10 physicians	74.0	50.3
Availability of fellowship program at work place	No	55.0	37.4
	Yes	89.0	60.5

In case 1, there were wide variations in the diagnostic procedures recommended by participants (Table 2). The majority of respondents recommended additional laboratory tests to the CBC test; SF and TIBC were the most frequent (70.7, 51%, respectively) recommended additional tests. However, only 15.6% of the respondents selected the “no other tests necessary” option. Moreover, 2% of respondents suggested blood film as another additional lab test not include in the list.

**Table 2:** Responses of participants to case 1

Case #1 A previously healthy 18-month-old male is referred to your clinic for evaluation of anemia. He was exclusively breastfed for 8 months, and since then has been receiving 1–1 ½ liter whole cow milk daily and limited iron-rich foods. The physical examination is normal except for pallor. His hemoglobin is 8.1 g/dL, RBC count 4 million/mm3, RDW 20%, and MCV 58 fL. No other laboratory tests were previously performed by the PCP.		
	Respondent answers	%
In addition to a CBC, which of the following tests would you routinely obtain when first seeing such a patient in your office (Select all that apply)?		
1	No other tests necessary	15.6
2	Reticulocyte count	28.6
3	Serum Ferritin	70.7
4	C-reactive Protein (CRP)	4.8
5	Serum Transferrin Saturation	15.0
6	Serum Iron	38.8
7	Hemoglobin Electrophoresis	15.6
8	Serum transferrin receptor	6.1
9	Reticulocyte hemoglobin content (CHr or Retic-He)	3.4
10	Blood lead level	0
11	Total iron binding capacity (TIBC)	51.0
12	Other (Please specify)	2.0
Which oral iron preparation would you recommend (assuming insurance and access are not problematic)?		
1	Ferrous sulphate (Fer-In-Sol®, Feromin®, Kdiron®)	77.6
2	Ferrousfumarate (Fumafer®,Ferretts®)	6.8
3	Ferrous gluconate (Ferrous gluconate®, Fergon®),	3.4
4	Iron (III)- hydroxide polymaltose (Ferose-F®, Ferose®)	10.2
5	Other (please specify)	2.0
Which factors contribute to your recommended oral iron preparation (Select all that apply)?		
1	Previous successful experience with it	51.0
2	Medical literature (published clinical studies involving that iron formulation)	44.9
3	Cost / Insurance	16.3
4	Taste / Tolerability	29.3
5	Practice / Recommendation of your partner(s)	16.3
6	Recommendation of the hematologist(s) with whom you trained	16.3
7	Other (please specify)	5.4
What total daily elemental iron dose would you recommend (Select one)?		
1	2–3 mg/kg	27.9
2	4–5 mg/kg	32.0
3	6 mg/kg	32.7
4	other (please specify)	6.8
5	Missing	0.6
If the patient’s hemoglobin was 6.1 g/d (rather than 8.1), what total daily elemental iron dose would you recommend (Select one)?		
1	No change	8.8
2	2–3 mg/kg	5.4
3	4–5 mg/kg	21.8
4	6 mg/kg	47.6
5	other (please specify)	15.6
6	Missing	0.8
If the patient’s hemoglobin was10.1 g/dL (rather than 8.1), what total daily elemental iron dose would you recommend (Select one)?		
1	No change	19.0
2	2–3 mg/kg	34.0
3	4–5 mg/kg	24.5
4	6 mg/kg	17.7
5	other (please specify)	4.1
6	Missing	0.7
How would you divide the total daily iron dose (Select one)?		
1	Once daily (QDay)	34.0
2	Divided into 2 doses (BID)	57.1
3	Divided into 3 doses (TID)	7.5
4	Other (please specify)	0.7
5	Missing	0.7
What is the hemoglobin value below which you would definitely recommend a blood transfusion (assuming the child looks “well compensated” with no co-morbidities) (Select one)?		
1	There is no hemoglobin below which I would definitely recommend a blood transfusion	21.8
2	3 g/dL	7.5
3	4 g/dL	2.0
4	5 g/dL	14.3
5	6 g/dL	40.1
6	Other (please specify)	11.6
7	Missing	2.7
Case #1 (continued) At a follow-up visit at 12 weeks, the patient’s hemoglobin is 12.2 g/dL, MCV 78 fL and ferritin 25 ng/mL, and his whole cow milk intake is limited.		
Would you recommend continued oral iron therapy (Select one)?		
1	No	31.3
2	Yes, 1–2 additional month of iron therapy	36.1
3	Yes, 3 or more additional months of iron therapy	25.9
4	Other (please specify)	4.1
5	Missing	2.6

Amongst the listed oral iron preparation, most (77.6%) of the respondents preferred treatment with ferrous sulfate; previous successful experience was the most (51%) cited reason. In terms of total daily iron dose, the respondents’ recommendations were equally distributed across the three listed choices.

Respondents were asked about total daily elemental iron dose they will recommend based on the degree of anemia severity. If the patient’s Hb was 6.1 g/dl (rather than 8.1 g/dl), 8.8% indicated that they would not change the dose. The majority (47.6%) of the respondents recommended a 6 mg/kg dose, whereas 5.4% would choose a 2–3 mg/dl dose. In case of patient’s Hb was 10.1 g/dl (rather than 8.1 g/dl), 19% indicated they would not change the dosage, whereas the majority (34%) would choose a 2–3 mg/dl dose and 24.5% of the respondents would choose a 4–5 mg/dl dose. In terms of dose frequency, the majority (57.1%) recommended 2 doses (BID) and 34% recommended a once daily dose (QDay).

In regard to recommended course of action for the Hb value below which blood transfusion should definitely be recommended, for well compensated children, wide variability in responses was found. Approximately 38% recommended blood transfusion when Hb value is 6 g/dl, whereas 21% responded that there is no Hb below which they would recommend a blood transfusion. Hb values from 3 to 5 g/dl were chosen by some respondents in different percentages; 7.5 to 14.3%.

As a continuation of this case scenario, participants were asked whether they will continue oral therapy at the 12th weeks visit if the patient’s hemoglobin is 12.2 g/dL, MCV 78 fL and ferritin 25 ng/mL and his whole cow milk intake is limited. Approximately 31% indicated that they will not continue iron therapy, 36.1% indicated that they will continue iron therapy 1–2 additional month and 25% they recommend continuation for a further 3 months or more, and 4% indicated other continuation periods.

In case scenario 2, majority of respondents recommended treatment with ferrous sulfate (76.2%) divided into two daily doses (62.6%). While 50.3% of respondents reported that patient’s daily dose should be based on weight, 49.7% recommended that dose should be based on number of tablets. For participants who reported that the daily dose will be based on the number of tablets, approximately 42.5% reported that they will choose 1 iron tablet daily, 53.4% reported that they will choose 2–3 iron tablets daily, and 4.1% reported other numbers of tablets. In terms of those who recommended daily dose should be based on the weight, 32.4% recommended a 2–3 mg/kg dose, 58.1% recommended a 4–5 mg/kg dose and 6.7% indicated other doses; Table 3.

**Table 3:** Responses of participants to case 2

Case #2 A previously healthy 13-year-old girl is referred to you for evaluation and treatment of anemia. She experienced menarche 2 years ago and has heavy menstrual periods. She has no evidence of a generalized bleeding disorder, and hormonal regulation has been initiated by her gynecologist. Her physical exam reveals a weight of 65 kg and mild pallor but is otherwise unremarkable. Her hemoglobin is 9.5 g/dL and MCV 65 fL. Other laboratory studies and her peripheral smear are consistent with iron deficiency. You again choose to treat with an oral iron product.		
Which oral iron preparation would you recommend (Select one)?		
1	Ferrous sulphate(Fer-In-Sol®, Feromin®, Kdiron®)	76.2
2	Ferrous fumarate (Fumafer®,Ferretts®)	7.5
3	Ferrous gluconate (Ferrous gluconate®, Fergon®)	4.8
4	Iron (III)- hydroxide polymaltose (Ferose-F®, Ferose®)	8.8
5	Other (please specify)	0.7
6	Missing	2.0
Would this patient’s daily dose be based on number of tablets daily or on weight (Select one)?		
1	Number of tablets daily	49.7
2	Weight-based dosing	50.3
Follow-up for persons who chose number of tablets daily: What total daily elemental iron dose would you recommend (Select one)?		
1	1 iron tablet daily	42.5
2	2–3 iron tablets daily	53.4
3	Other (please specify)	4.1
Follow-up for persons who choose weight-based dosing: What total daily elemental iron dose would you recommend (Select one)?		
1	2–3 mg/kg	32.4
2	4–5 mg/kg	58.1
3	Other (please specify)	9.5
How would you divide the total daily iron dose (Select one)?		
1	Once daily (QDay)	28.6
2	Divided into 2 doses (BID)	62.6
3	Divided into 3 doses (TID)	4.8
4	Other (please specify)	2.0
5	Missing	2.0
Case #2 (continued) The 13 year old patient returns to your clinic 4 weeks after the initial visit. Her last several menstrual periods have been light. She reports that she took the iron medication for several days after her visit with you but then stopped it because of gastrointestinal symptoms. Her laboratory studies are similar to the previous visit. You change to another oral iron preparation, but she continues to have recurrent gastrointestinal symptoms and no improvement in her laboratory studies. You describe parenteral iron treatment options, and she is interested.		
Which iron preparation would you recommend (Select one)?		
1	Intravenous iron dextran (Cosmofer®)	42.9
2	Intravenous iron saccharate (Ferosac®)	32.7
3	Continued oral iron therapy	14.3
4	Other (please specify)	10.1

In continuation to the case scenario 2, respondents were asked regarding parenteral iron treatment they would recommend in case there is no response to oral iron. Most (42.9%) respondents indicated IV iron dextran. While 32.7% of prefer IV iron saccharate, 13.4% reported that they would continue oral iron therapy. Moreover, 10.9% of respondents specified some other treatments in the free text comment instead of listed options, including seeking the advice of dietitian, refer to hematologist, and admission for observation.

Correct responses on different questions related to diagnosis and management of IDA are represented in Table 4. Participants were less likely to consider no other laboratory test necessary beyond CBC 23(15.6%). However, SF had the highest proportion of the preferred lab tests; 71.2%.

**Table 4:** correct responses on different questions related to diagnosis and management of IDA

Q		*N* (%)
1	In addition to a CBC, which of the following tests would you routinely obtain when first seeing such a patient in your office?*	
	-No other tests necessary	23(15.8)
	-Reticulocyte count	42(28.8)
	-Serum Ferritin	104(71.2)
	-Serum Transferrin Saturation	22(15.1)
	-Serum Iron	57(39)
	-Serum transferrin receptor	9(6.2)
	-Total iron binding capacity (TIBC)	75(51.4)
	-Other (Blood film)	3(2.1)
2	Which oral iron preparation would you recommend (assuming insurance and access are not problematic)?	129(87.8)
3	Which factors contribute to your recommended oral iron preparation?	141(95.9)
4	What total daily elemental iron dose would you recommend?	100(68)
5	If the patient’s hemoglobin was 6.1 g/dL (rather than 8.1), what total daily elemental iron dose would you recommend?	116(78.9)
6	If the patient’s hemoglobin was10.1 g/dL (rather than 8.1), what total daily elemental iron dose would you recommend?	91(61.9)
7	How would you divide the total daily iron dose?	146(99.3)
8	What is the hemoglobin value below which you would definitely recommend a blood transfusion (assuming the child looks “well compensated” with no co-morbidities)?	49(33.3)
9	Would you recommend continued oral iron therapy?	95(64.6)
10	Which oral iron preparation would you recommend?	125(85)
11	Would this patient’s daily dose be based on number of tablets daily or on weight?	74(50.3)
12	Follow-up for persons who choose weight-based dosing: What total daily elemental iron dose would you recommend?	43(58.1)
13	How would you divide the total daily iron dose?	146(99.3)
14	Which iron preparation (parenteral) would you recommend?	48(32.7)

*Represent list of all correct diagnostic tests

Majority of the respondents recognized the appropriate oral iron preparation (87.8%) and related factors that should guide their recommendations of the optimum oral iron preparation; 95%. While the vast majority of participants (99.3%) could properly divide the total daily iron dose, 68% were able to identify the total daily elemental iron dose. Percentages of the correct answers related to which total daily elemental iron dose they would choose if the Hb was 6.1 g/dl or 10.1 g/dl (rather than 8.1 g/dl) were 78.9, 61.9%, respectively. However, they were less likely to know the Hb value below which they would recommend blood transfusion (33.3%). Approximately 64% of the participants identified the correct answer associated with continued iron therapy when the patient improved and lab result showed normal Hb, MCV, and ferritin values. For case scenario 2, a high proportion identified the correct oral iron preparation (85%), and only half of them were able to indicate the correct daily dose. However, 58.1% of them had positive knowledge about weight-based dose. While 99.3% of participants could know the correct division of the total daily iron dose, only 32.7% of them identified the optimum parenteral iron treatment.

Differences in overall median scores of correct answers by demographic and professional characteristics are represented in Table 5. Overall scores differed significantly by professional qualification (*P* = 0.007) and type of current work setting (*P* = 0.043).

**Table 5:** Differences in overall scores by demographic and professional characteristics

Characteristics		Overall Score		
		Median	(Q1,Q3)	*p*
Gender	Female	68	(63–74)	0.217*
	Male	68	(63–79)	
Professional qualification	GP	63	(63–74)	0.007†
	Family medicine	68	(61–76)	
	Pediatrician	68	(63–79)	
	Pediatric Hematologist	84	(71–89)	
Professional rank	Assistant consultant	74	(63–79)	0.413†
	Associated consultant	68	(63–74)	
	consultant	68	(63–76)	
	Staff physician	63	(63–74)	
	Resident	68	(58–74)	
Type of current work setting	primary care	63	(63–74)	0.043*
	Tertiary care	68	(63–79)	
Years in practice since fellowship	0–5 years	68	(58–74)	0.848†
	6–10 years	68	(63–79)	
	11–15 years	68	(61–76)	
	> 15 years	68	(63–79)	
No. of co-worker pediatricians	1–2 physicians	63	(58–74)	0.188†
	3–5 physicians	68	(63–68)	
	6–10 physicians	74	(68–79)	
	> 10 physicians	68	(63–79)	
Availability of Fellowship program at work place	No	68	(63–74)	0.813*
	Yes	68	(63–79)	

* Mann-Whitney Test †Kruskal-Wallis Test Q1-Q3: first and third quartiles

Robust regression analysis for factors associated with overall score is represented in Table 6. In univariate analysis, professional qualification and type of current work setting were the only two variables statistically significantly associated with overall score. In the final multivariate analysis model, the only variable that was independently significantly associated with increased overall scores was professional qualification (*P* = 0.017). Score of Pediatric Hematologists was 14.98 times higher than score of GPs (β = 14.98, CI 6.81–23.15, *P* < 0.0001). Compared with scores of participants who work in primary care, score of participants who work in tertiary care was 3.63 times higher (β = 3.63, CI -0.03-7.28, P 0.05).

**Table 6:** Factors associated with overall score

Factor	*N* (%)	Univariate analysis		Multivariate analysis	
		β (95% CI)	*p*	β (95% CI)	*p*
Professional qualification					
Pediatric hematologist	8(5.4)	14.98(6.81,23.15)	< 0.0001	13.71(2.48,24.95)	0.017
Pediatrician	77(52.4)	2.87(−1.02,6.77)	0.15	1.77(−6.05,9.59)	0.66
Family medicine	12(8.2)	2.69(−4.20,9.58)	0.44	2.66(−4.30,9.58)	0.45
General practitioner	50(34)	1		1	
Type of current work setting					
Tertiary	76(51.7)	3.63(−0.03,7.28)	0.05	1.26(−6.40,8.92)	0.75
Primary	71(48.3)	1		1	

Based on findings of the above detailed regression analysis, further analyses were carried out to fully describe differences in scores both in the diagnostic and therapeutic domains questions by professional qualifications and type of current work setting. Consistently, the proportion of those achieved highest score of correct answers were pediatric hematologists, compared with respondents with other professional qualifications. General Practitioners had the lowest proportion. Moreover, participants who work in tertiary care had significantly higher proportion of those achieved highest score of correct answers compared with those who work in primary care.

## Discussion

To the best of our knowledge, this is the first study conducted in the Arabian Gulf region to assess the diagnostic and therapeutic modalities employed by physicians for the management of pediatric IDA. The study findings show wide variations in practices and recommendations regarding the proper diagnosis and treatment of pediatric IDA in KSA by physicians involved in management and care. This reflects lack of local guidelines for management of this widely prevalent medical condition. National guidelines are urgently needed. Further, the study identifies some healthcare practitioners who might benefit from further training to improve their pediatric IDA management skills. Namely, physicians working in primary care setting, GPs, family medicine practitioners and pediatricians.

Research work on diagnostic criteria and therapeutic modalities employed by physicians for pediatric IDA is sparse. While previous studies focused on IDA epidemiology [1, 7, 8, 12, 29], diagnosis [24, 29–31], treatment [24, 32], or prevention [28, 29, 33], findings of our study and another study conducted in U.S by Powers et al. [17] were the only two studies to demonstrate a wide variation among physicians in IDA diagnosis and treatment. However, compared with Powers et al. study, the present study adopted a unique approach. The total score we calculated in this study, based on participants responses to different questions, allowed us to identify various sub-optimal practices regarding diagnosis and treatment of pediatric IDA.

The documented wide variations in this study regarding the diagnostic criteria for IDA worth consideration. Many of the patients managed at primary care facilities or referred to tertiary hospitals undergo unnecessary tests in addition to the CBC [31]. The study finding that only 15.6% of participants in the present study recommended that no additional laboratory tests necessary beyond the CBC concur with findings of a study conducted in the U.S. by Powers J et al. [17]They also found a similar percentage of 15%.

AAP guidelines recommend that patient found anemic on initial screening should undergo confirmative testing for dubious IDA that include measuring TfR1 concentration, CHr or Ret-He, and/or SF with CRP [29]. However, the AAP does not indicate that neither CHr cannot differentiate between IDA and thalassemia nor TfR1 cannot differentiate between IDA and hemolytic anemia [34]. Further, these tests are not readily available in all healthcare centers, particularly in resource-limited countries. Therefore, making a clinical decision based on such tests might not be viable. In this study, most participants do not use a specific guidelines to confirm diagnosis of IDA. In the aforementioned U.S. study which surveyed pediatric hematology/oncology physicians, most participants did not employ the AAP’s approach [17]. Powers et al. recommended that CBC, peripheral blood smear and reticulocyte count along with serum iron, SF and TIBC usually can be used to establish the diagnosis of IDA [23]. While such tests were chosen by over 50% of Powers et al. study participants, SF and TIBC were selected by approximately half of our participants.

In regard IDA treatment, elicited responses indicate that over half of our study participants base their therapeutic decision on their previous experience than on evidence. Almost all published literature recommends a dose of 3–6 mg/kg/day of iron as an efficient IDA treatment [24, 30]. However despite using 6 mg/kg/day being the overwhelming preference for pediatric hematology oncology specialists, there is no evidence base for such choice. Many studies have shown treatment success with low dosages. A randomized trial in Ghana compared patients to 40 mg of iron, or almost 3 mg/kg/day either as a single dosage or in three divided dosages, found similar success in both groups [32].Another study including 90 elderly patients compared three daily doses of iron (15 mg, 50 mg, and 150 mg). After 60 days, in all three groups, the increases in Hb concentration and SF were similar. This suggests that low oral dosage of iron treatment could be as effective as high dosage. Furthermore, in the lowest dose groups, the rates of dropout and adverse effects were lower [35].However, the optimal duration for oral iron supplementation has not been specified yet but previous studies recommend 3–6 months supplementation [25, 26, 36]. Canadian Pediatric Surveillance Program recommends 6 mg/kg/day of oral iron dose for 3–4 months [44]. It has been suggested that Iron stores are refilled after continuation of iron therapy for 2–3 months [36].

Responses of participants showed a marked variability regarding the duration of continued oral iron treatment when the patient’s Hb, MCV and ferritin became normal. This is comparable to Powers et al. study [17]. However, in this study, 50.3% of respondents recommended patient’s daily dose should be based on weight and 49.7% based on number of tablets compared with 64 and 36% respectively in that study. Although the Food and Drug Administration (FDA) approved many safe and effective parenteral iron preparations for adult patients and children who could not responded to oral iron therapy, only 14% of participants in the present study recommended this course of action. Approximately, of 170 iron therapies used to treat and prevent IDA, ferrous sulfate was most effective and least cost compared with other oral iron treatments [37].Currently, it is the most frequent treatment for IDA’s patients [23].

Study participants received very low scores on other two items of the questionnaire. In the question regarding the Hb value below which they would definitely recommend a blood transfusion (assuming the child looks “well compensated” with no co-morbidities), only 33.3% identified the correct strategy. Indeed, there is no globally agreed threshold in terms of the blood transfusion, and hence, some physicians recommend blood transfusion based on symptoms and clinical condition regardless of the Hb values [45]. Further, regarding which parenteral iron preparation should be used, only 32.7% of the participants indicated the correct IV preparation. Relevant studies indicate that IV administration of iron dextran increases Hb in 7–14 days [38], and iron saccharate (iron sucrose) and ferric gluconate increases the Hb after 7 days [39].Clinical trials conducted to detect AEs after administering iron sucrose [40], ferric gluconate [41] and iron dextran [42]reported a frequency of 36, 35, and 50% among study participants, respectively. Another study conducted by Sayyad et al. found that administration of the iron sucrose was not associated with serious AEs [46]. Further, iron sucrose and ferric gluconate found to be better than iron dextran in term of higher bio-availability and lower occurrence of anaphylaxis which is considered life-threatening [43]. Consequently, using iron dextran was restricted in the U.S. due to its multiple AEs, such as anaphylactic reactions [47, 48]. However, these low proportions of correct answers maybe explained by the fact that most GPs, family physicians, and some pediatricians transfer their patients to a pediatric hematologist in cases of transfusion or IV iron preparation. This concurs with our findings that higher scores were achieved by pediatric hematologists and those working in tertiary care setting. In the final multivariate robust regression analysis model pediatric hematologists score of was 14.98 times higher than that of general practitioners.

This study has some limitations. The study represents assessment of GPs, family physicians, pediatricians and pediatric hematologists who work in single healthcare facility in KSA and might not be generalizable to practitioners in other national healthcare facilities. No other similar local data are available to confirm the findings of this study. Although the response rate of participation is high (88%), responses of those declined to complete the questionnaire might be different from those retained in this study. This might have introduced, albeit minimum, ascertainment bias. However, those who declined participation are not likely to be different from those who participated in the study. The assessment of responses was based on local expert opinion and limited international published studies and might not reflect a global consensus on management of pediatric IDA.

## Conclusion

A wide variation exists in diagnostic and treatment modalities employed by physicians for pediatric IDA in KSA. This is further exasperated by lack of evidence-based guidelines for the optimal management of this widely diagnosed medical condition. These guidelines are urgently needed. Large scale clinical trials and prospective studies are needed to better inform these guidelines and programs. The study identifies certain healthcare professionals that might benefit from intervention programs aimed at maximizing optimal management of IDA patients.

## Data Availability

The datasets used and/or analyzed during the current study are available from the corresponding author on reasonable request.

## Abbreviations

AEs: Adverse events
APP: American Academy of Pediatrics
CBC: Complete blood count
CHr: Reticulocyte Hemoglobin Content
CRP: C-reactive protein
FDA: Food and Drug Administration
GP: General practitioner
Hb: hemoglobin
ID: Iron Deficiency
IDA: Iron deficiency anemia
IV: Intra venous
KSA: Kingdom of Saudi Arabia
MCV: Mean Corpuscular Volume
QDay: Once daily dose
RBC: Red blood cells count
SF: serum ferritin
TfR1: Transferrin receptor 1
